# Using Listening Rooms for Qualitative Data Collection in Medical Science Education Research

**DOI:** 10.1007/s40670-026-02875-5

**Published:** 2026-09-04

**Authors:** Emily Green, Beth Lawry, Vanessa Armstrong, Alison I. Graham

**Affiliations:** 1 Newcastle University (School of Medicine), Newcastle upon Tyne, UK; 2 Newcastle University (School of Biomedical, Nutritional and Sport Sciences), Newcastle upon Tyne, UK

**Keywords:** Qualitative research, Methodology, Research methodology, Research techniques, Feedback (learning)

## Abstract

Listening Rooms are a qualitative data collection method, wherein two participants have a discussion without a researcher present. The conversation is guided by prompts provided by the researcher. This approach can elicit more candid, honest accounts of experiences than may occur when participants communicate directly with researchers, which can be advantageous in medical science education contexts. This article outlines practical tips for getting the most out of using Listening Rooms in medical science education research, including guidance on deciding whether listening rooms are appropriate for a particular project, advice regarding prompt design, and logistical, safety and wellbeing factors to consider.

## Introduction

Qualitative research has grown in popularity since it was introduced into the medical science education field in the 1980s [1–3]. Traditionally, qualitative data collection has relied on interview or focus group techniques, or analysis of written data. These approaches are generally logistically straightforward, allow the researcher to guide the conversation to ensure relevant data is collected, and can provide detailed insight into the experiences of participants [4]. However, there are limitations of these, including risk of “social desirability bias” [5] and facilitators presenting a source of distortion [6–8]. In interviews, the dynamic between researcher and participant may not be conducive to open and comfortable discussion, particularly where there are existing power dynamics (for example, in an educator-student scenario) [9], or where the researcher does not belong to the same group or community as the participant (for example, an able-bodied researcher exploring the experiences of disabled participants) [10]. Whilst it is often best practice for an interview or focus group to be led by someone unknown to participants, this is not always possible. For these reasons, there is growing interest in alternative approaches to qualitative data collection, including poetry [11], “love and breakup letters” [12], and art-based methodologies [13]. These alternative approaches allow participants to provide insights beyond simply describing their experiences. However, direct interaction with the researcher is often still required, and some of these approaches lack interaction with other participants. Here, we discuss an alternative qualitative data collection approach, which removes the researcher from direct conversation with the participant, whilst still allowing interaction and discussion between participants, to facilitate open, honest and insightful discussions. This approach is known as Listening Rooms (LRs).

### What are Listening Rooms?

LRs involve two participants having a conversation, without the researcher present. The discussion is guided by prompts or cue cards provided by the researcher and is recorded for transcription and analysis. LRs are an emerging approach, developed by Heron and Parkin [14–16], who have used the method to explore student experiences, including that of minoritized groups. The two participants may be randomly paired, or may be existing friends (one inviting the other to participate), the latter described as using “*friendship as a method*” [14]. LRs have been identified as valuable for gaining honest feedback in institutional settings, by eliciting more detailed and constructive suggestions than traditional qualitative methods [17]. Previous work has established LRs as an effective alternative method for qualitative data collection, particularly when candid disclosure is less likely to occur in direct conversation with the researcher.

By removing direct researcher interaction, LRs can elicit rich, honest and meaningful discussion through discovery of shared experiences when participants are strangers but come from the same culture or community, or through the existing relationship and rapport of friendship pairs [14–16, 18]. The authors experiences of using LRs has found they can elicit open storytelling and honest accounts of experience by removing the pressure of speaking directly to a researcher, giving freedom to participants explore phenomena in ways that are meaningful to them, potentially covering topics and interpretations which may not have been anticipated by the researcher. In this way, LRs have some similarities to non-participant observation approaches, wherein the researcher passively observes participant interactions whilst avoiding influencing the environment or discussions [19]. LRs differ by physically removing the researcher from the data collection interaction, whereas in observational approaches, participants are aware of researcher presence, which may inadvertently influence participant responses. Despite the researcher not being present to observe in real-time, LRs do offer some control over participant conversations through carefully prepared prompts or cue-cards. In this way, LRs lie closer to traditional data collection approaches of interviews or focus groups, by aiming to guide participants to discuss particular phenomena, whereas non-participant observation aims to explore factors beyond words (such as behaviors and social interactions) [19]. LRs provide a middle-ground, between hands-off observational approaches, and more researcher-involved approaches such as interviews or focus groups. Of course, removing the researcher from the conversation does reduce the level of control over the direction of discussion, which is a possible drawback and needs to be weighed against the possible benefits and limitations of greater participant freedom when deciding whether to use the LR approach. LRs are not a covert data collection method, as participants are fully aware that they are being recorded. Of course, this may lead to some tempering of their responses, but the authors have found that participant discussions through LRs are still open and honest, and likely more so than if participants were interviewed directly. It is challenging to ethically justify covert approaches to data collection; LRs provide an alternative where participants do not have to speak directly to researchers, but are still aware their data is being collected.

LRs may confer particular advantages in medical science education research contexts. Students are time-poor and strategically focused [20, 21]. This is especially true of medical students, who are often under particular pressure due to the competitive culture of medical school [22, 23], creating greater risk of feelings of imposter syndrome [24] and magnifying power imbalances between staff and students. This can prevent student participants feeling able to share honest views and opinions directly with researchers or may create discomfort with sharing experiences of challenges with groups of fellow students. LRs can help mitigate power imbalances by removing the researcher from direct communication with participants and allowing the use of friendship pairs rather than conversations with large groups of unfamiliar participants. Issues with imposter syndrome and power dynamics extend beyond university [25], meaning LRs may also be a powerful method for data collection involving external partners, industry, healthcare professionals or patients, where existing power dynamics between colleagues, or between partners and those they perceive as being part of the institution, create barriers to more open storytelling. LRs have been described previously in higher education contexts [14–16] but have not been widely discussed or operationalized in medical science education research, despite the possible benefits of the approach in this field. Here, we outline best-practice guidance for using LRs in medical sciences education research, based on existing literature and our own experiences of using the approach.

### Case Studies

The authors have not identified any existing publications describing using LRs in the context of medical science education, meaning much of their learning was taken from the original use of LRs by Heron and Parkin [14–16] and through the authors’ own experiences. The authors have all recently utilized LRs for qualitative data collection in medical science education research projects. One of the authors (EG) used LRs to explore neurodivergent medical student experiences of gross anatomy teaching and learning, recruiting 20 participants to 10 LRs, using a mixture of randomly-paired participants, and participants who had chosen their own partner. Over 17 h of recordings were generated, transcribed, and analyzed using a hermeneutic phenomenological approach [26]. The remaining authors (BL/VA/AG) used LRs to investigate experiences of students within the Faculty of Medical Sciences who were from low-socioeconomic backgrounds, LGBTQ+, parents or carers, and/or had a disability or long-term illness, recruiting six participants to three listening rooms (project ongoing), with transcriptions thematically analyzed [27] using an iterative approach. In both projects, LRs were selected after careful consideration of various data collection methods, as it was felt that the power dynamic of the researcher being an educator and possibly not belonging to the same demographic or minoritized group as the students may cause participants to feel uncomfortable sharing their views in a traditional interview or focus group.

The article aims to introduce LRs to other medical science education researchers who may find it an illuminating and powerful approach, and to provide best-practice guidance including potential pitfalls and key points to consider. The tips are grounded in existing literature and the key lessons learned from our use of LRs in the above case studies. These case studies will be used to exemplify and illustrate the tips provided.

## Tips for Effective Use of LRs in Medical Science Education Research

1. Ensure LRs are appropriate for your project

Alignment between the research question(s), methodology and methods is important in any research project [28]. The research design and the appropriateness of methods to address the research question also underpin rigor and trustworthiness of qualitative research [29]. Therefore, researchers should carefully consider the methodological and logistical advantages and limitations of LRs compared to other data collection approaches. LRs may be particularly beneficial in medical science education contexts, where the competitive and intense environment [22, 23] can create pressure for students to share socially desirable views. Similarly, LRs can be valuable where participants may fear judgement or repercussions for sharing honest opinions directly with peers or with researchers who are also educators [9]. The approach can also be beneficial when working with participants who may be less comfortable sharing their views with a stranger, by using friendship pairings. The use of friendship pairs means there is existing rapport and familiarity between participants, which can save time compared to traditional approaches where initial stages of the focus group or interview may need to be used to ”break the ice” and build comfort between participants and researchers. LRs are useful when exploring a phenomenon, culture, or community that the researcher is unfamiliar with or may not be part of, by allowing participants to explore the topics in question on their own terms. For this reason, LRs are best suited to exploring broader phenomena and more open-ended research questions. The reduced control over the conversation may limit discussion of highly nuanced topics, meaning LRs are not appropriate where very specific areas must be explored. In addition, participants are unsupervised during LRs, meaning they may be inappropriate for highly sensitive or emotive topics.

2. Take a careful and iterative approach to prompt development

**Fig. 1:**
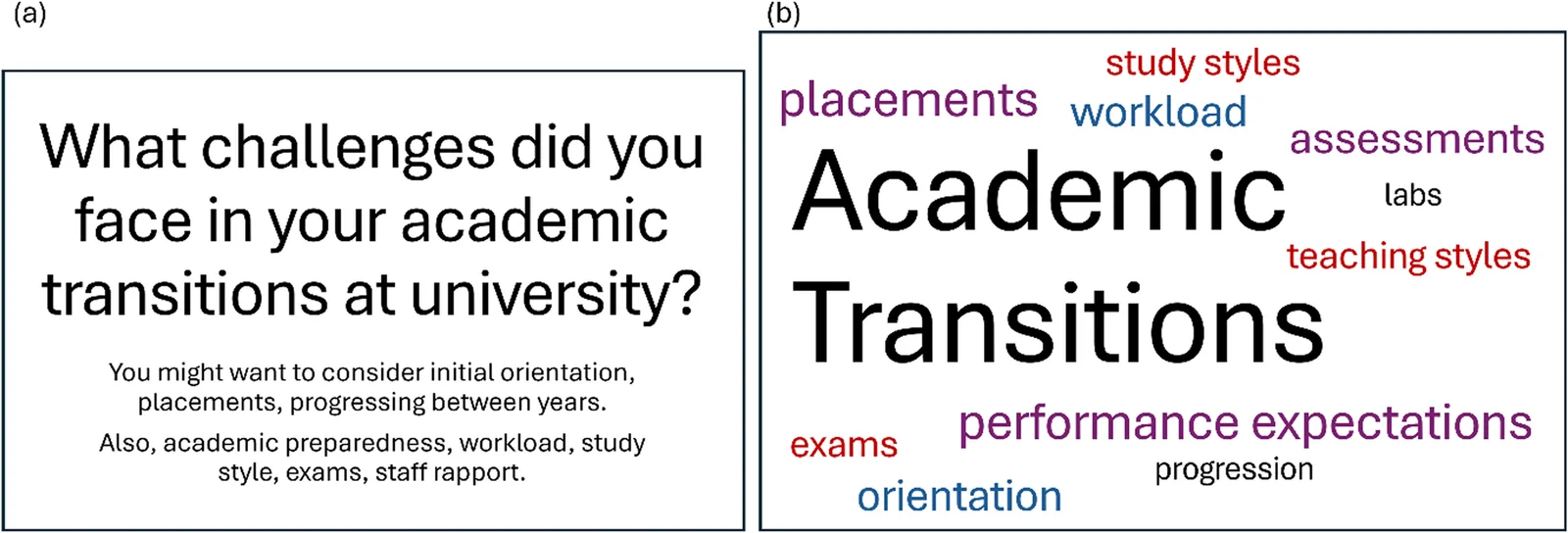
Progression of LR prompts developed throughout the authors’ (BL/VA/AG) project. **a** Initially, participants were provided with a choice of prompts with questions and sub-headings and encouraged to select those that most resonated with them. However, participant feedback from the initial sessions indicated that the magnitude of choice was overwhelming. **b** Based on this, prompts were modified into “word cloud”-style cards, which proved much more successful in ensuring participants felt sufficiently guided throughout the LR despite the lower word count of the revised prompts

The prompts or cue cards provide the main input the researcher has into the direction of the LR conversation. These should therefore be subject to thoughtful and thorough development. Prompts should be grounded in the research question(s) and aims, incorporating relevant conceptual frameworks or theory [30]. One of the authors (EG) used the neurodiversity paradigm as a conceptual framework [31]. The remaining authors (BL/VA/AG) used co-design principles [32, 33] to develop prompts together with students. Involving members of the culture or community being explored in the design of LR prompts can help ensure participant voices are heard at all stages of the study design. Both EG and BL/VA/AG discussed and reviewed their initial prompts with key community members who had knowledge or experience of the phenomena of interest, to provide refinement and differing viewpoints on how the prompts may be interpreted. In both projects, prompts were also amended iteratively throughout data collection, by listening back to the recorded conversation and modifying prompts if needed. Piloting prompts, or developing these iteratively as the LRs take place, ensures they are being interpreted as intended and that relevant discussion is occurring. Iterative prompt development aligns well with grounded theory approaches [34], where moving between data collection and analysis informs theoretical development. Developing prompts throughout analysis also provides opportunities for researchers to probe emerging issues or concepts that have arisen during initial LRs. BL/VA/AG initially provided a choice of three prompt packs to their participants, each containing a choice of cards (Fig. 1a). Feedback from participants in initial pilot testing indicated that the quantity of prompts and text was overwhelming. In response, BL/VA/AG worked with students to reduce the number of prompts, creating accessible and visual word clouds as an alternative (Fig. 1b). EG found that prompt development had been largely successful, with only minor tweaks to some wording required during the data collection process.

Researchers should also consider how LR outcomes will be used when developing prompts. For example, EG used the challenges identified through LRs to inform co-creation workshops with students, to develop possible solutions. This meant it was critical that prompts encouraged discussion of actionable, practice-informing topics. Offering prompts in a specific order or at random is a decision for researchers based on their topic of the study. Accessibility of prompts should be considered, for example by considering readability for dyslexia [35, 36].

3. Be creative

**Fig. 2:**
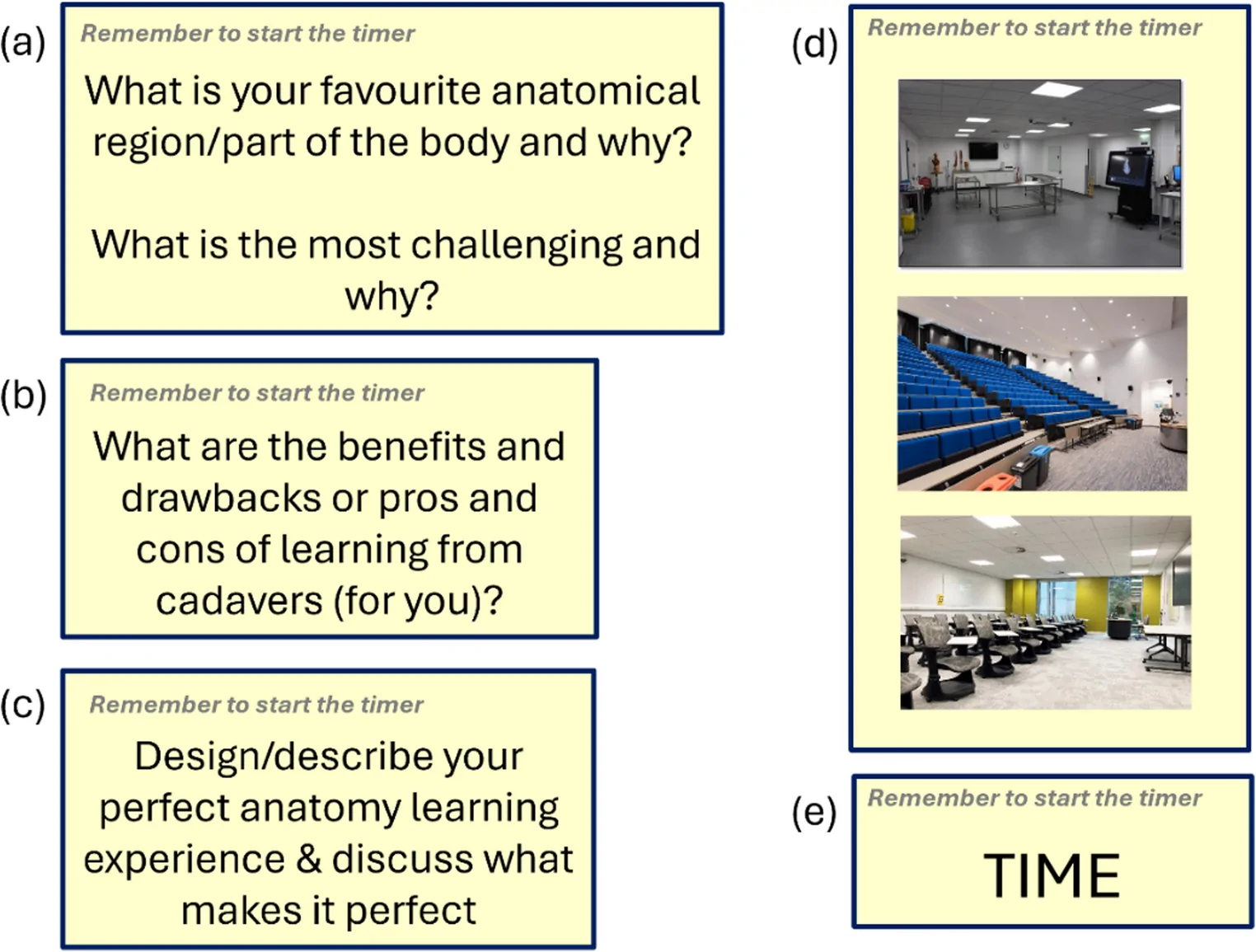
Examples of prompts used in the authors’ (EG) project. **a** This prompt was numbered ‘1’ and was designed as a low-stakes area of discussion to act as an icebreaker between participants. **b**, **c** Statements or questions provide clear guidance and ensure conversation remains on topic. **d** Image prompts, in this case showing different learning environments, were found to be a powerful approach for evoking memories of experiences. **e** Single-word prompts may carry the risk of causing confusion in participants but also provide broad scope for interpretation which can bring about discussion of unanticipated (but relevant) topic areas. It is important to test prompts or be open to iteratively developing these throughout data collection to ensure they are being interpreted as intended

Prompts do not have to be limited to questions or statements (Fig. 2a, b and c). Researchers should be creative in considering how to elicit genuine, emotive discussion of authentic lived experiences. Artwork, poetry, audio or video prompts could be explored [37, 38]. Images are effective for evoking memories [39]; EG found participants frequently responded by describing how images “brought back memories”, or “took them back” to a moment in time (Fig. 2d). EG also used single-word prompts (Fig. 2e). These sometimes led to initial confusion from participants, but then almost always led to insightful, relevant discussion despite initial uncertainty. Single-word prompts are open to interpretation, providing space for participants to respond in ways relevant to them and their experience, which aligns well with phenomenological methodologies [40]. A detailed briefing (Tip 9) can help prepare participants for unexpected prompts.

4. Consider safety, wellbeing and ethical factors

Participant safety and wellbeing are essential considerations, particularly as participants are unsupervised during the LR. As with all research projects involving human participants, ethical approval should be sought prior to commencing the study. Here, guidance and ideas are provided regarding how to support participant wellbeing and ethical use of LRs. A participant briefing is crucial (Tip 9); expectations, logistics and ground rules should be clearly explained to foster an environment of open conversation where both participants are given the opportunity to speak. The briefing is a crucial safeguarding step, and should make clear to participants that the content of the discussion should remain confidential. The room used should consider accessibility and comfort (e.g. furniture, temperature, background noise) and allow the researcher to be nearby if issues arise, but not able to listen in to the conversation “live” (for example, positioned in a next door or nearby room to the LR location). It is good practice to provide all participants with wellbeing resources and information within the LR room, and/or provide a de-briefing that includes wellbeing information and signposting [41]. These wellbeing resources should be iteratively developed throughout data collection, particularly if unexpected issues or conversation topics arise. As discussed in Tip 1, ensuring LRs are appropriate for the topic being discussed and that sensitive or highly emotional areas are avoided is a key step for ensuring participant safety and wellbeing. Regardless of the topic, participants should be informed they can skip any prompts they do not wish to discuss, and should be made aware of the location of the researcher during the LR to allow them to seek support if any distress is caused or if they have any queries. Recordings should be reviewed promptly by the researcher (listened to within a week of the LR taking place), to identify and address any safeguarding concerns and to support ongoing prompt development and analysis. If safeguarding or wellbeing concerns are identified, the participant(s) should be contacted and provided with additional, relevant wellbeing resources.

5. Be comfortable with lack of control

Expect the unexpected! Interviews and focus groups allow researchers to intervene and control the direction of conversation and balance of contributions as discussion takes place. When using LRs, this control is significantly reduced, meaning the conversation may move into unanticipated or unintended areas, which can be a powerful way to gain understanding of participant lifeworlds and lived experiences [14, 40]. LRs give power to participants to control the flow of discussion and topics of conversation, allowing them to explore areas that resonate most with them. However, this reduced control may be uncomfortable for researchers more experienced in traditional approaches. The authors encourage an open mindset; in our projects, despite researchers not being present during discussions, participants stayed on topic and rich, highly relevant data was collected. If data collected using LRs highlights areas that researchers would like to explore or probe further, iterative prompt development (Tip 2) can address this, or LRs could be followed up with other approaches (e.g. focus groups, interviews) to provide further clarity if needed.

Without the researcher being physically present during the conversation, it is not possible to intervene or probe in real-time when data collection is no longer productive or when sufficient depth has been achieved in relation to the research question. The only recourse the researcher has in these situations is to adapt the prompts or briefing documents for future LRs. Whilst the absence of adaptive strategies introduces challenges for rigor and feasibility, this must be balanced against the benefits of free-flowing conversations between pairs. Furthermore, it is important to note that recent discourse within qualitative research has rejected the idea of saturation or data sufficiency [42], instead arguing for the idea of “information power” [43, 44] or the “amount of information a sample holds”, which is partly related to “quality of dialogue” LRs have scope to produce richer data wherein both sides of the conversation are open to analysis, and may therefore result in data with greater information power. This is a further argument for ensuring the quality and efficacy of the LR prompts (Tip 2), as controlling off-topic or irrelevant discussion as much as possible is important for maintaining dialogue quality.

6. Be mindful of logistics

LRs may present additional logistical challenges compared with traditional methods. Coordinating availability for two participants, the researcher, and an appropriate location can be challenging. Mottershaw et al. [18] provide useful pragmatic guidance on managing LR logistics. Contingency plans should be in place if one participant is absent, such as offering an interview alternative. LRs suit both in-person and online settings. In-person sessions allow the researcher to ensure there is provision of additional measures to support participant comfort, including comfortable chairs, refreshments and sensory aids or fidget toys [45], but require recording equipment and transcription software. Online LRs improve accessibility for individuals who may not be able to attend in-person (such as parents or carers) and recording and transcription software is often integrated into online meeting platforms which can reduce costs. Flexibility is ideal, allowing participants to choose the format that best suits them [46]. The transcription and analysis process may be time-consuming for LRs as, without facilitation, there is heightened risk of off-topic discussion, lengthy monologues, participants speaking over each other, and challenges distinguishing between two voices.

7. Decide on chosen or random pairings

Deciding whether pairings are chosen by the participants, selected randomly by the researcher, or both, will be guided by the research topic and practical considerations. Existing familiarity between chosen pairs can facilitate increased disclosure, and richer, more insightful responses [14], and “collaborative recall” means friendship pairs may remember more than paired strangers [47, 48]. However, allowing participants to choose their partner introduces some ethical complexity as researchers must ensure both participants have freely consented to participate. Ensuring both participants separately sign up and complete consent forms with ample opportunity to ask questions is important. Random pairings are advantageous when recruiting from potentially socially-isolated groups, allowing individuals to participate even if they are unable to identify a friend to join them. However, additional time may be needed to build rapport and gain familiarity before free-flowing conversation occurs. In the authors’ projects, participants were given the option to choose their partner or opt for a random pairing. The authors found that both pairing types led to generation of insightful, rich data, even when participants were unfamiliar to each other. Prompt design can be used to create icebreakers, by using a lower-stakes question as the first prompt that is easier to interpret and discuss (Fig. 2a). While unfamiliarity may lead to superficial dialogue, this was not observed in our projects, where random pairings still generated meaningful discussion.

8. Consider your timings

Imposing a time limit on the LR is pragmatic and respectful of participants’ time. The authors allocated one hour per LR, as in previous studies [14, 16]. This was felt to balance pragmatism with time for adequate discussion, but flexibility is helpful if participants feel the allocated time is insufficient, by considering additional time or a follow-up LR. Introducing a time limit per prompt, which will relate to the number of prompts used, should be considered. Based on previous work [14, 16], the authors used six prompts, with a 10-minute limit per prompt (a timer was provided for participants). Using more prompts with less time per prompt would help to focus exploration into specific areas but could limit depth or overwhelm participants. Fewer prompts with more time for each allows breadth and richness of discussion, but risks participants drifting off topic. Researchers need to balance pragmatism with the goals of their study when making decisions regarding total time for the LR, and time allocations per prompt.

Some researchers may reject the idea of time limits per prompt entirely, as these could risk ”cutting off” or curtailing relevant discussion. Open-ended timing offers a more organic approach, allowing participants to allocate time for prompts depending on what resonates with them. However, limitless time per prompt may cause participants to become engrossed in conversation and lose track of time, therefore not completing all prompts, or alternatively they may rush through the prompts and lose depth of discussion. Imposing a time limit per prompt ensures each prompt is sufficiently discussed, as participants can be encouraged to re-visit prompts and reflect further if they finish early.

9. Provide a detailed participant briefing

As well as information on the study and use of data to ensure informed consent can be given, a briefing should provide guidance on LR logistics, establish ground rules, share wellbeing information (Tip 4) and allow time for questions. Logistical guidance may include:

- Confidentiality and anonymity – participants should be instructed not to share content from the discussion outside of the LR.
- Timings and timers (if used).
- What to expect from the prompts, particularly any that are less straightforward to interpret (Tip 3).
- Procedures if participants need a break or to leave early.
- Provision of pre-identified accessibility requirements.
- How to contact the researcher during the LR.
- Mobile phone/device use.
- What to do if participants finish discussing a prompt or struggle to interpret it (e.g. re-read the prompt, attempt to continue discussing, or move on).
- Whether participants can add final thoughts at the end.

Ground rules are particularly important for randomly-paired participants, and may include: establishing a psychologically safe, respectful, and non-judgmental space; topics to avoid; reiterating the importance of confidentiality; and whether there are any details or circumstances the participants wish to disclose beforehand. A clear briefing can help mitigate possible problems and should be reviewed throughout the study.

10. Collect participant feedback

Collecting participant feedback on the LR experience and process can be informative, especially when researchers are new to LRs. This can be achieved informally (e.g. a conversation with participants after the LR) or more formally (e.g. a brief structured questionnaire). Researchers will need to decide whether it is best to collect comments immediately after the LR, or whether it would be most productive to do this some time after to allow participants to reflect on the full experience. Participants should be encouraged to give feedback on all aspects of the process (from initial recruitment, to giving informed consent, to their experience of the LR itself). Feedback is vital for effective prompt development and to ensure accessibility and comfort. BL/VA/AG made significant modifications to their prompts based on initial feedback from participants, resulting in more effective data collection and enhanced participant experience.

## Strengths, Limitations and Further Considerations

The principal strength of LRs is the removal of the researcher from the data collection interaction. This removes pressure on participants to ”perform” or share views they believe the researcher is looking for, encouraging honest and open sharing of experiences. This is important when power dynamics between the researcher and the participant may impede participants from sharing their honest views, particularly in education research contexts, where educators may be carrying out research with student participants. This is also powerful in situations where researchers are studying cultures or communities they are not a part of; researchers may fail to anticipate certain aspects of a phenomenon and therefore not encourage discussion of this in a traditional interview or focus group. LRs allow participants from similar backgrounds to share common experiences and explore phenomena in ways that are resonant to them, potentially diverging into topics that were not expected by the researcher. Accessibility is another key driver for selecting LRs, as the approach suits participants who may find it challenging to engage in traditional interviews and focus groups, and would prefer to speak with a friend or peer as opposed to a researcher. Data collected through LRs is often rich and detailed, particularly as both sides of the conversation are relevant to analysis, as opposed to an interview, where one side of the dialogue is the researcher posing questions or probing. Existing research and our own experiences of LRs have used the approach with only two participants; there may be scope to explore LRs with three or four participants. However, the authors’ view is that this should be considered with caution, as doing so could not only introduce further complexity in the transcript, but also has the potential to reduce the main benefits of the approach in creating a safe space for participants to share their honest views. More than two participants could also increase the chance that not everyone will speak and contribute equally.

However, when using LRs, there are possible limitations. The main drawback is the reduced researcher control over the direction of the conversation, meaning it is possible that participants may spend time discussing irrelevant topics. Careful prompt development and use of timings can help ensure participant discussions remain focused, but there is no scope for addressing tangents or distractions contemporaneously. Similarly, researchers cannot probe or more deeply explore issues raised at the point of discussion, these can only be revisited in future LRs through modified or additional prompts. Additionally, the rich, two-sided conversations can be lengthy and complex to transcribe and analyze. It is important to be mindful of the topics that may be explored through the LR conversation, and sensitive or highly emotional areas should be avoided, and wellbeing support provided for participants.

There are several other factors that researchers should also consider if planning to use LRs. LRs are often appropriate and impactful when working with participants from minority groups or marginalized communities [49, 50]. It can be powerful to involve these communities within the study design process itself; development of LR prompts lends itself well to co-production approaches. When working with minoritized or marginalized groups, it is important to consider how researchers might then “close the loop” in the analysis and dissemination process, by providing feedback to participants on findings, outputs and impact [51]. This can empower individuals by allowing them to see how their voices have been listened to and recognize the resulting changes. One way this can be achieved is by actively involving key players in the data analysis process, as seen in the round-table analysis used by Heron and Parkin [16]. However, LRs are a data collection approach only and may therefore align with various qualitative analysis techniques depending on the research paradigm and methodology used. For example, if an iterative approach to prompt development is taken, this may align well with grounded theory methodologies [34].

Within current LR and most qualitative research, reflexivity is critical, requiring researchers to continually examine their own position and assumptions throughout the research process [14, 16, 52]. Reflexivity is a key contributor to trustworthiness and rigor in qualitative research [29, 53]. Researchers must consider how their roles and identities might shape their data collection or interpretation when using LRs, ensuring they attempt to mitigate potential biases and enhance the authenticity of data. One of the author’s (EG’s) projects utilized a hermeneutic phenomenological approach to analysis, as hermeneutics acknowledge the role of the researcher in meaning-making and interpretation of participant accounts of lived experiences [40, 29, 53]. Regardless of the analysis approach used, findings should be shared in accessible formats that ensure participants can access and understand project outputs (e.g. audiovisual summaries to help communicate written abstracts [54]), as marginalized groups have historically encountered barriers when engaging in research (both as participants and researchers). Creating openly available outputs as well as flexible and inclusive opportunities for participation can help contribute to dismantling systemic inequalities.

## Conclusions

LRs are a powerful approach for qualitative data collection and can mitigate some of the limitations of traditional methods. In the context of medical science education research, the approach can act to address potential power imbalances between educators and students, particularly within medical or health professions education where these imbalances may be magnified. LRs are particularly useful for exploring the experiences of marginalized groups, where the researcher may not belong to the community or culture under investigation. LRs provide agency for participants to explore phenomena in ways that are relevant and resonant for them. Removing direct interaction with the researcher can elicit more truthful responses and therefore more rich and relevant data. As discussed, the approach may be limited by the removal of direct researcher control over the discussion, but this can be mitigated by provision of carefully designed prompts, and is a limitation that is often outweighed by the benefits of unrestricted peer-to-peer conversation.

LRs provide an interesting alternative or adjunct to traditional focus groups or interviews, and when used mindfully, can open up areas of discussion that may not have been previously anticipated. Flexibility and open-mindedness are helpful, allowing researchers to realize the full potential of the method in gathering honest, open, and often unexpected accounts. The tips provided here support medical science education researchers in incorporating this approach mindfully into their practice.

## Data Availability

Data sharing not applicable to this article as no datasets were generated or analyzed during the current study.

## References

[1] Smith Iii AC, Noblit GW. The idea of qualitative research in medical education. Teach Learn Med. 1989;1(2):101–8.

[2] Farghaly A. Comparing and Contrasting Quantitative and Qualitative Research Approaches in Education: The Peculiar Situation of Medical Education. Educ Med J. 2018;10(1):3–11.

[3] Boet S, Sharma S, Goldman J, Reeves S. Review article: Medical education research: an overview of methods. Can J Anesthesia/Journal canadien d’anesthésie. 2012;59(2):159–70. 10.1007/s12630-011-9635-y 22215522

[4] Paradis E. The Tools of the Qualitative Research Trade. Acad Med. 2016;91(12):e17. 10.1097/ACM.0000000000001393 27655057

[5] Bergen N, Labonté R. Everything Is Perfect, and We Have No Problems: Detecting and Limiting Social Desirability Bias in Qualitative Research. Qual Health Res. 2020;30(5):783–92. 10.1177/1049732319889354 31830860

[6] Stokes D, Bergin R. Methodology or methodolatry? An evaluation of focus groups and depth interviews. Qualitative Market Research: Int J. 2006;9(1):26–37.

[7] Smithson J. Using and analysing focus groups: Limitations and possibilities. Int J Soc Res Methodol. 2000;3(2):103–19.

[8] Lopez V, Whitehead D. Sampling data and data collection in qualitative research. In: Nursing & midwifery research: methods and appraisal for evidence-based practice. 4th ed. Elsevier Mosby; 2013. p. 123–40.

[9] Seale J, Gibson S, Haynes J, Potter A. Power and resistance: Reflections on the rhetoric and reality of using participatory methods to promote student voice and engagement in higher education. J Furth High Educ. 2015;39(4):534–52.

[10] Jain NR, Nimmon L, Bulk LY. How to … bring a JEDI (justice, equity, diversity and inclusion) lens to your research. Clin Teach. 2024;21(1):e13660. 10.1111/tct.13660 37874114

[11] Brown MEL, Kelly M, Finn GM. Thoughts that breathe, and words that burn: poetic inquiry within health professions education. Perspect Med Educ. 2021;10(5):257–64. 10.1007/s40037-021-00682-9 PMC8505582 34472010

[12] Laughey WF, Brown MEL, Liu A, Dueñas AN, Finn GM. Love and breakup letter methodology: A new research technique for medical education. Med Educ. 2021;55(7):818–24. 10.1111/medu.14463 33529431

[13] Fraser KD, al, Sayah F. Arts-based methods in health research: A systematic review of the literature. Arts Health. 2011;3(2):110–45.

[14] Heron E. Friendship as method: reflections on a new approach to understanding student experiences in higher education. J Furth High Educ. 2020;44(3):393–407.

[15] Parkin H, Heron E. Innovative methods for positive institutional change: The Listening Rooms Project and student and staff ‘voice’. Educational Developments. 2022;23(3):13–7.

[16] Heron E, Parkin H. Listening works: using the Listening Rooms methodology to explore diversity. J Learn Dev High Educ. 2023;(26):2–14.

[17] Hulme A, Evans M. Evaluating the Impact of Embedded Academic Language and Literacies Provision in a School of Education and School of Music. ESP Today. 2025;13(2):419–40.

[18] Mottershaw S, Gent S, Clark S, Ugiagbe-Green I, Awolowo F, Owolade F. Listening Rooms Toolkit v2 2025. Available from: https://sway.cloud.microsoft/uPgMLVr5OcqpLUbw

[19] Ciesielska M, Boström KW, Öhlander M. Observation Methods. In: Ciesielska M, Jemielniak D, editors. Qualitative Methodologies in Organization Studies: Volume II: Methods and Possibilities. Cham: Springer International Publishing; 2018. pp. 33–52.

[20] Wolters CA, Brady AC. College Students’ Time Management: a Self-Regulated Learning Perspective. Educational Psychol Rev. 2021;33(4):1319–51.

[21] Wladis C, Hachey AC, Conway KM. It’s About Time, Part II: Does Time Poverty Contribute to Inequitable College Outcomes by Gender and Race/Ethnicity? AERA Open. 2024;10:23328584241237971.

[22] Lempp H, Seale C. The hidden curriculum in undergraduate medical education: qualitative study of medical students’ perceptions of teaching. BMJ. 2004;329(7469):770–3. 10.1136/bmj.329.7469.770 PMC520997 15459051

[23] Shaw S, Anderson J. Coping with medical school: an interpretive phenomenological study. The Qualitative Report. 2021;26(6):1864–80.

[24] Khan M. Imposter syndrome—a particular problem for medical students. BMJ. 2021;375:n3048. 10.1136/bmj.n3048 34887245

[25] LaDonna KA, Ginsburg S, Watling C. Rising to the Level of Your Incompetence: What Physicians’ Self-Assessment of Their Performance Reveals About the Imposter Syndrome in Medicine. Acad Med. 2018;93(5):763–8. 10.1097/ACM.0000000000002046 29116983

[26] Ajjawi R, Higgs J. Using Hermeneutic Phenomenology to Investigate How Experienced Practitioners Learn to Communicate Clinical Reasoning. The Qualitative Report. 2015.

[27] Braun V, Clarke V. Using thematic analysis in psychology. Qualitative Res Psychol. 2006;3(2):77–101.

[28] Cleland J, Durning SJ, editors. Researching Medical Education. 2nd ed. Chichester, West Sussex, UK: John Wiley & Sons Ltd; 2023.

[29] Cypress BS. Rigor or Reliability and Validity in Qualitative Research: Perspectives, Strategies, Reconceptualization, and Recommendations. Dimens Crit Care Nurs. 2017;36(4):253–63. 10.1097/DCC.0000000000000253 28570380

[30] Varpio L, Paradis E, Uijtdehaage S, Young M. The Distinctions Between Theory, Theoretical Framework, and Conceptual Framework. Acad Med. 2020;95(7):989–94. 10.1097/ACM.0000000000003075 31725464

[31] Shaw SCK, Brown MEL, Jain NR, George RE, Bernard S, Godfrey-Harris M et al. When I say … neurodiversity paradigm. Med Educ. 2024;59(5):466–8. 10.1111/medu.15565 PMC11976190 39563003

[32] Sanders EBN, Stappers PJ. Co-creation and the new landscapes of design. CoDesign. 2008;4(1):5–18.

[33] Clark AT, Ahmed I, Metzger S, Walker E, Wylie R. Moving From Co-Design to Co-Research: Engaging Youth Participation in Guided Qualitative Inquiry. Int J Qualitative Methods. 2022;21:16094069221084793.

[34] Watling CJ, Lingard L. Grounded theory in medical education research: AMEE Guide 70. Med Teach. 2012;34(10):850–61. 10.3109/0142159X.2012.704439 22913519

[35] Shaw SCK, Anderson JL. Twelve tips for teaching medical students with dyslexia. Med Teach. 2017;39(7):686–90. 10.1080/0142159X.2017.1302080 28298166

[36] British Dyslexia Association. Dyslexia friendly style guide 2023. Available from: https://www.bdadyslexia.org.uk/advice/employers/creating-a-dyslexia-friendly-workplace/dyslexia-friendly-style-guide

[37] Stelling H, Ueda M, Tilby-Jones F, Brown M, Burford B, Fisher J et al. From passive participants to proactive partners: How to engage students in the design, delivery and development of research using the principles of co‐production. Clin Teach. 2024;21(6):e13804. 10.1111/tct.13804 39218616

[38] Philion R, St-Pierre I, Bourassa M. Accommodating and supporting students with disability in the context of nursing clinical placements: A collaborative action research. Nurse Educ Pract. 2021;54:103127. 10.1016/j.nepr.2021.103127 34237511

[39] Glaw X, Inder K, Kable A, Hazelton M. Visual Methodologies in qualitative research. Int J Qual Methods. 2017;16(1):1–8.

[40] van Manen M. Researching lived experience: Human science for an action sensitive pedagogy. 2nd ed. London, Ontario, Canada: Althouse; 1997.

[41] O’Connor R. Working-class imposterism in the law school: I always have this in the back of my head that I’m expected to fail. Law Teacher. 2025;59(2):217–41.

[42] Braun V, Clarke V. To saturate or not to saturate? Questioning data saturation as a useful concept for thematic analysis and sample-size rationales. Qualitative Res Sport Exerc Health. 2021;13(2):201–16.

[43] Malterud K, Siersma VD, Guassora AD. Sample Size in Qualitative Interview Studies: Guided by Information Power. Qual Health Res. 2016;26(13):1753–60. 10.1177/1049732315617444 26613970

[44] Roberts S-J, Saylor MA, Ivynian S, DiGiacomo M. Data saturation and information power: sample size in qualitative research—when is enough and how to assess? Eur J Cardiovasc Nurs. 2026;25(5):1069–74. 10.1093/eurjcn/zvag046 41693424

[45] Brown K. Running an autism-friendly product focus group: a case study from Rare Birds 2023. Available from: https://www.autism.org.uk/advice-and-guidance/professional-practice/running-an-autism-friendly-product-focus-group-a-c

[46] Heath J, Williamson H, Williams L, Harcourt D. It’s just more personal: Using multiple methods of qualitative data collection to facilitate participation in research focusing on sensitive subjects. Appl Nurs Res. 2018;43:30–5. 10.1016/j.apnr.2018.06.015 30220360

[47] Marion SB, Thorley C. A meta-analytic review of collaborative inhibition and postcollaborative memory: Testing the predictions of the retrieval strategy disruption hypothesis. Psychol Bull. 2016;142(11):1141–64. 10.1037/bul0000071 27618544

[48] Vredeveldt A, van Deuren S, van Koppen PJ. Remembering with a friend or a stranger: comparing acquainted and unacquainted pairs in collaborative eyewitness interviews. Memory. 2019;27(10):1390–403. 10.1080/09658211.2019.1662052 31500512

[49] Wolstenholme C, Sen J. Using Listening Rooms as a method to engage and understand male ethnic minority student views of seeking university mental health support. J Public Mental Health. 2025;25(2):187–96.

[50] Awolowo IF, Dosumu O, Olawoyin F, Abidoye A, Ajao O. The impact of personalised mentorship on the academic, personal, and professional development of black students in UK higher education. Stud High Educ. 2025;1–18. 10.1080/03075079.2025.2591802

[51] Powell L. We’re Not Your Empathy Exercise: Reclaiming Co-Production in Neurodivergent Research and Practice. Neurodiversity. 2025;3:27546330251363393.

[52] Olmos-Vega FM, Stalmeijer RE, Varpio L, Kahlke R. A practical guide to reflexivity in qualitative research: AMEE Guide 149. Med Teach. 2022:45:1–11. 10.1080/0142159X.2022.2057287 35389310

[53] Stahl NA, King JR. Expanding Approaches for Research: Understanding and Using Trustworthiness in Qualitative Research. J Dev Educ. 2020;44(1):26–8.

[54] Reupert A. Promoting research through video abstracts. Adv Mental Health. 2017;15(1):1–3.

